# Learning styles of medical students, general surgery residents, and general surgeons: implications for surgical education

**DOI:** 10.1186/1472-6920-10-51

**Published:** 2010-06-30

**Authors:** Paul T Engels, Chris de Gara

**Affiliations:** 1 Department of Surgery, University of Alberta, Walter C. Mackenzie Centre, University of Alberta Hospital, 8440-112 Street NW, Edmonton, Alberta T6G 2R7, Canada

## Abstract

**Background:**

Surgical education is evolving under the dual pressures of an enlarging body of knowledge required during residency and mounting work-hour restrictions. Changes in surgical residency training need to be based on available educational models and research to ensure successful training of surgeons. Experiential learning theory, developed by David Kolb, demonstrates the importance of individual learning styles in improving learning. This study helps elucidate the way in which medical students, surgical residents, and surgical faculty learn.

**Methods:**

The Kolb Learning Style Inventory, which divides individual learning styles into Accommodating, Diverging, Converging, and Assimilating categories, was administered to the second year undergraduate medical students, general surgery resident body, and general surgery faculty at the University of Alberta.

**Results:**

A total of 241 faculty, residents, and students were surveyed with an overall response rate of 73%. The predominant learning style of the medical students was assimilating and this was statistically significant (p < 0.03) from the converging learning style found in the residents and faculty. The predominant learning styles of the residents and faculty were convergent and accommodative, with no statistically significant differences between the residents and the faculty.

**Conclusions:**

We conclude that medical students have a significantly different learning style from general surgical trainees and general surgeons. This has important implications in the education of general surgery residents.

## Background

Educating surgeons is an age-old tradition that has existed since the development of surgery and is now entrenched within our modern Hippocratic Oath[1]. Modern surgical education has been crafted and shaped by visionaries such as Halsted who have helped evolve the historical model of apprenticeship into the current organized system of surgical education that we know as Residency[2]. However, the demographics of the next generation of surgeons[3] and the methods by which they are trained are rapidly evolving[4], especially with the evolution of surgical simulation[5]; it has recently been suggested that the role for this historic apprenticeship no longer exists in the era of modern surgical education[6].

Current surgical trainees now originate from a diverse educational, cultural, ethnic, and gender background[2], and are responsible for developing skills not only in the role as a medical expert, but in the role as a professional, scholar, health advocate, manager, collaborator, and communicator[7]. These changing demographics and demands call for the implementation of more effective and efficient training programs.

In order to train surgeons effectively and efficiently, it is important to consider not only what they are learning but how they are doing so. Consideration of the way that a trainee learns is becoming increasingly important[8]. Accumulating evidence suggests that surgical trainees have specific learning styles[5,9-11] and identifying and focusing on these has the potential to improve the delivery of surgical education. While other studies have examined the relationship of learning styles to specific aspects of education[5,9], we sought to assess the general learning styles of pre-surgical trainees, surgical trainees, and practicing surgeons, to better assess for differences between them and any evidence of evolution of learning style.

Learning style is the process by which a person understands and retains information, thereby gaining knowledge or skills[12]. Many models and measures of learning styles have been described in the literature[8], including Kolb's Learning Inventory[13] and Gardner's Multiple Intelligence Theory[9,14]. Kolb's Learning Style Inventory, has been applied to evaluating trainees and practitioners in the fields of Internal Medicine[12], Pediatrics[15], General Surgery[10,11] and Anesthesia[16] over a span of several decades. The Kolb model has been criticized for not applying to all situations, for paying insufficient attention to the process of reflection, taking little account of cultural-based learning differences, and the relationship of learning processes to knowledge[17]. Nevertheless, no model is perfect at present and the Kolb model serves as a well-established model which allows the comparisons of learning styles across medical specialties and between training levels, and its experiential basis is particularly relevant to the apprenticeship model of surgical training.

The Kolb Learning Cycle first introduced by David Kolb in 1984 and is based on the principle of the learning cycle which all individuals use to acquire knowledge[13]. The model divides the learning cycle into four stages: experiencing--immersing oneself in the doing; reflection--reviewing what has been done; conceptualization--interpreting the event; and planning--predicting subsequent actions. This learning cycle is based on the concept that the more often a task is reflected on the more often there is the opportunity to modify and refine one's efforts. The logic of the learning cycle is to make many small and incremental improvements. The model utilizes a learning style inventory as a method of measuring learning dimensions and styles of individual and team members. An individual's preferred learning style can be assessed by answering a relatively short survey which asks the participant to rank certain statements about learning in the order as they apply to themselves. An individual's preferred learning style is categorized as Diverging, Assimilating, Converging, or Accommodating. Recognition of these different learning styles within individuals and educational systems can improve the efficiency of learning[8].

The various learning styles and their characteristics within the Kolb model are described as follows[13]. An individual with a diverging learning style exhibits strength in imaginative ability and performs well in situations that call for "brainstorming", and enjoys broad cultural interests; characteristically found in those from humanities and liberal arts backgrounds. Those with assimilating learning styles enjoy creating theoretical models and place importance in theory and logic, and are less interested in people and more concerned with abstract concepts; characteristically found in research and planning departments. A person with a converging learning style enjoys the practical application of ideas and uses hypothetical-deductive reasoning, remaining relatively unemotional and preferring to deal with things rather than people; characteristically found in engineers and those with narrow technical interests. An individual with an accommodating learning style enjoys doing things and being involved in new experiences, takes risks, and excels where one must adapt to specific immediate circumstances but may be seen as impatient and pushy; characteristically found in action-oriented jobs such as marketing or sales.

To date, studies in general surgery have shown that the predominant learning style of surgical trainees is Converging and Accommodating[10,11], with possible significant implications for academic performance and trainee selection. A study by Windsor et al, although focusing on multiple intelligence learning styles and surgical simulation, also suggested a role for learning styles in trainee selection[9]. However, there is a paucity of information about whether this is the result of self-selection or learning style evolution as residents progress through training, nor any large-scale studies on the predominant learning style of the surgical faculty themselves.

The purpose of our study was to define the predominant learning styles of medical students, general surgery residents, and general surgery faculty, and to show any differences between these groups.

## Methods

**Figure 1:**
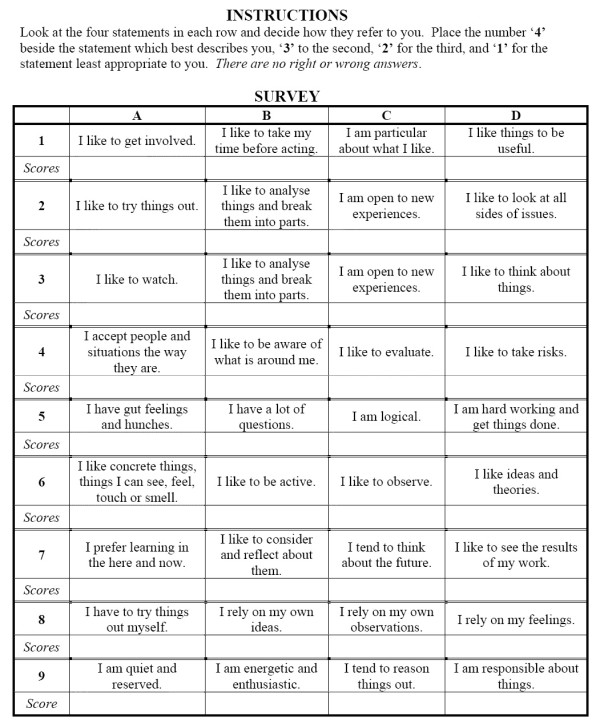
**Kolb Learning Style Inventory administered to cohorts**.

An established evaluative tool, the Kolb Learning Style Inventory[13] (see Figure 1), was administered to three groups at the University of Alberta: the entire 2^nd ^year pre-clinical undergraduate medical class (N = 157); general surgery residents (N = 40); general surgery faculty (N = 44). The survey and an explanatory covering letter were distributed to the groups via email or in person, with follow-up reminder emails sent to improve the response rate. Each participant was offered to receive the result of their survey as well as an explanatory sheet on learning styles. The data were compiled into a Microsoft Excel^® ^(Microsoft Corporation, USA) spreadsheet. It was analyzed for the dominant learning style within each group as well as each individual residency year using the two-sample z test for two independent proportions. This project received approval by the Health Research Ethics Board at the University of Alberta.

## Results

**Figure 2:**
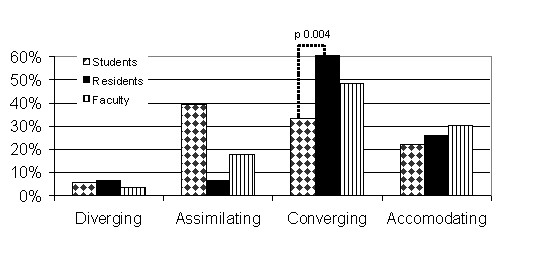
Learning Styles of Students, Residents, and Faculty.

**Figure 3:**
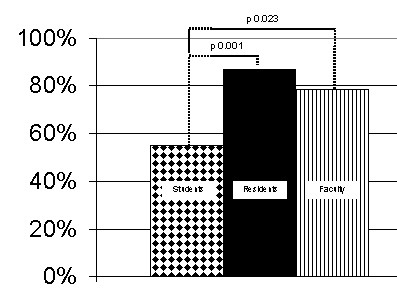
Prevalence of Converging or Accommodating Learning Styles among Students, Residents, and Faculty.

Overall survey response rate was 73% (177/241). Amongst the individual groups, response rates were 75% for the students, 78% for the residents, and 64% for the faculty. The learning styles within each group are shown in Figure 2. The groups were analyzed with two-sample z test for two independent proportions. Within the residency cohort, the only significant difference between the years was the first year residents having a significantly (p < 0.003) less prevalent presence of the combined converging and accommodating learning style than the rest of the resident cohort. When examined for single learning style differences, the students had a statistically significant (p < 0.004) smaller presence of the converging learning style than the residents. When combining the accommodating and converging learning styles into a single category there were statistically significant differences between the groups, as illustrated in Figure 3. There was a difference in the prevalence of converging or accommodating learning styles between the students and residents (55% vs. 79%, p = 0.001) and students and faculty (55% vs. 87%, p = 0.023). There was no statistically significant difference between the residents and faculty (79% vs. 87%, p = 0.383).

## Discussion

Understanding how people learn is important in order to improve the teaching and learning environment[8], and most critically to actually improve an individual's learning. The ability to constructively modify one's behaviour depends on how well we combine our experiences, reflection, conceptualization, and planning to make improvements. Recognizing an individual's strengths and preferred learning style in this process will allow the tailoring of the learning experience to that person and increase the efficiency with which something is learned, with obvious positive effects on time, utilization, and outcome.

Our study shows that the predominate learning styles of the general surgery residents and general surgery faculty at the University of Alberta are convergent and accommodative, consistent with previous studies[10,11]. We found no significant differences in learning styles between the residents and faculty. We did, however, find statistically significant differences in the learning styles of the undergraduate medical students as compared to the general surgery residents and faculty, with the predominant learning style amongst the students being assimilating. Comparatively, the assimilating learning style was recently found to be the most common style among internal medicine residents[12]. These observations have a number of potential implications.

As surgical residents have different inherent learning styles than medical students, educational techniques used in undergraduate medical education should not be arbitrarily applied to surgical training without further validation. Efforts should be made to gear the surgical education to the learning style of surgical residents. Furthermore, despite the majority of trainees and faculty having similar learning styles, there exists a minority (> 10%) of trainees whose learning style remains different from the group. While tailoring surgical education techniques to these learning styles, in general, should serve to increase the learning efficiency and effectiveness within this group, in particular, this will not serve each individual within the group. Individual tailoring of an educational program to one's learning style may be necessary as mismatches between instructor and learner is a potential learning obstacle[8].

For example, a resident with a converging learning style prefers the practical application of ideas and prefers hypothetical-deductive reasoning to solve problems. Ideal learning situations may include making multiple and diverse patient-care decisions, and assessing and evaluating available clinical literature, for which activities such as oral examinations with multiple scenarios and presenting rounds to an audience of peers on a specific topic may be beneficial to training. A resident who has an accommodating learning style enjoys becoming involved and taking risks as well as solving problems in a trial-and-error manner. Ideal learning situations may include opportunities to be the operating surgeon and exposure to surgical emergencies, for which training on surgical simulators and rotations in trauma and acute care surgery may improve his or her training.

The transition point of the learning style as a medical student to surgical trainee is not entirely clear. There appears to exist a difference in learning styles between the first year residents and the more senior residents, with the first year residents being more likely to have a diverging or assimilating learning style. However, the difference in learning styles is most pronounced between the student and resident cohorts. Thus, the mechanism of selection most likely takes place during or prior to the residency application process. In an effort to better understand any self-selection process that may take place, we sought to assess all the applicants to the University of Alberta General Surgery Residency Program by asking them to complete the learning style inventory immediately after their interview. Unfortunately, we did not receive any responses from this group. Obtaining this information may help answer the question of whether applicants to general surgery have already self-selected themselves based on their learning style or whether the applicant selection committee is responsible for such selection. We know that medical students choose careers based on income, prestige, debt, mentors, and clerkship experiences among many factors[18,19]. It is plausible that in the course of deciding on their career path that medical students align themselves with the specialty they most identify with and having a similar learning style may play an important factor.

Our study is limited by a number of features. Our response rate of 73%, although excellent for such a survey-style study, still leaves significant room for reporting-bias. The accommodating and converging learning styles were combined for analysis as this was done in prior studies[10] and we wished to be able to directly compare our results. The Kolb learning inventory is based on a model and has all the inherent limitations of that model.

## Conclusions

Medical education is constantly evolving. The implementation of work-hour restrictions on training programs has forced a re-examination of training methods and demands better and more efficient ways to train surgeons. The pressure for improved professionalism, communication and collaboration as well as patient and personal safety issues demand that all aspects of surgical education be examined, including how we learn. In order for surgical education to be optimized information needs to be gathered on how exactly young surgeons are selected and trained in order to know how we can do it better. Our study shows important differences in learning styles between undergraduate medical students and general surgery residents and faculty, consistent with an expanding body of knowledge in the literature. Acknowledgement of these differences and concerted efforts to make use of them are imperative to improve the training of future surgeons.

## Competing interests

The authors declare that they have no competing interests.

## Authors' contributions

PTE carried out the data acquisition, analysis, and interpretation, and drafted the manuscript. CDG participated in the design of the study, interpretation of the data, and revision of the manuscript. All authors read and approved the final manuscript.

## Pre-publication history

The pre-publication history for this paper can be accessed here:

http://www.biomedcentral.com/1472-6920/10/51/prepub
